# Characteristics and outcomes of antiretroviral-treated HIV-HBV co-infected patients in Canada?

**DOI:** 10.1186/s12879-019-4617-8

**Published:** 2019-11-21

**Authors:** Urvi Rana, Matt Driedger, Paul Sereda, Shenyi Pan, Erin Ding, Alex Wong, Sharon Walmsley, Marina Klein, Deborah Kelly, Mona Loutfy, Rejean Thomas, Stephen Sanche, Abigail Kroch, Nima Machouf, Marie-Helene Roy-Gagnon, Robert Hogg, Curtis L. Cooper, The Canadian Observational Cohort (CANOC) Collaboration

**Affiliations:** 1 School of Epidemiology and Public Health, University of Ottawa, Ottawa, ON K1G 5Z3 Canada; 2 College of Osteopathic Medicine, Michigan State University, East Lansing, MI 48824 USA; 3 Department of Medicine, University of Ottawa, Ottawa, ON K1H 8M5 Canada; 4 BC Centre for Excellence in HIV/AIDS, Vancouver, BC V6Z 1Y6 Canada; 5 Regina Qu’Appelle Health Region, Regina, SK Canada; 6 University Health Network, Toronto, ON M5G 2C4 Canada; 7 Research Institute of McGill University Health Centre, Montreal, QC H3H 2L9 Canada; 8 Memorial University of Newfoundland, Saint John’s, NL A1C 5S7 Canada; 9 Maple Leaf Medical Clinic, Toronto, ON M5G 1K2 Canada; 10 Clinique Medicale l’Actuel, Montreal, QC H2L 4P9 Canada; 11 Department of Medicine, University of Saskatchewan, Saskatoon, Saskatchewan S7N 5E5 Canada; 12 The Ontario HIV Treatment Network, Toronto, ON M4T 1X3 Canada; 13 Clinique de Médicine Urbaine du Quartier Latin, Montreal, QC H2L 4E9 Canada

**Keywords:** Co-infection, Hepatitis B, HIV, Prevalence

## Abstract

**Background:**

Hepatitis B (HBV) and Human Immunodeficiency Virus (HIV) share common risk factors for exposure. Co-infected patients have an increased liver-related mortality risk and may have accelerated HIV progression. The epidemiology and demographic characteristics of HIV-HBV co-infection in Canada remain poorly defined. We compared the demographic and clinical characteristics and factors associated with advanced hepatic fibrosis between HIV and HIV-HBV co-infected patients.

**Methods:**

A retrospective cohort analysis was conducted using data from the Canadian Observational Cohort (CANOC) Collaboration, including eight sites from British Columbia, Quebec, and Ontario. Eligible participants were HIV-infected patients who initiated combination ARV between January 1, 2000 and December 14, 2014. Demographic and clinical characteristics were compared between HIV-HBV co-infected and HIV-infected groups using chi-square or Fisher exact tests for categorical variables, and Wilcoxon’s Rank Sum test for continuous variables. Liver fibrosis was estimated by the AST to Platelet Ratio Index (APRI).

**Results:**

HBV status and APRI values were available for 2419 cohort participants. 199 (8%) were HBV co-infected. Compared to HIV-infected participants, HIV-HBV co-infected participants were more likely to use injection drugs (28% vs. 21%, *p* = 0.03) and be HCV-positive (31%, vs. 23%, *p* = 0.02). HIV-HBV co-infected participants had lower baseline CD4 T cell counts (188 cells/mm_3_, IQR: 120–360) compared to 235 cells/mm_3_ in HIV-infected participants (IQR: 85–294) (*p* = 0.0002) and higher baseline median APRI scores (0.50 vs. 0.37, *p* < 0.0001). This difference in APRI was no longer clinically significant at follow-up (0.32 vs. 0.30, *p* = 0.03). HIV-HBV co-infected participants had a higher mortality rate compared to HIV-infected participants (11% vs. 7%, *p* = 0.02).

**Conclusion:**

The prevalence, demographic and clinical characteristics of the HIV-HBV co-infected population in Canada is described. HIV-HBV co-infected patients have higher mortality, more advanced CD4 T cell depletion, and liver fibrosis that improves in conjunction with ARV therapy. The high prevalence of unknown HBV status demonstrates a need for increased screening among HIV-infected patients in Canada.

## Background

Approximately 40 million individuals are HIV-infected and an estimated 350 million people are infected with HBV (WHO, 2014). While the prevalence of HBV in Canada is decreasing due to immunization programs, it remains a public health issue partly as a consequence of the high prevalence among Canadian immigrants originating from HBV-endemic areas [1]. Other key at-risk groups in Canada include men who have sex with men (MSM), people who inject drugs (PWID), and Indigenous populations [1–4].

Due to shared routes of transmission including sexual contact, body fluid exposure, needle sharing, and vertical transmission, HIV-HBV co-infection is common [5, 6]. Liver disease resulting from HBV infection is a leading cause of non-AIDS related morbidity and mortality among HIV-infected individuals on antiretroviral therapy (ARV) [7–10]. Co-infection with HIV is associated with an increased risk of HBV chronicity, cirrhosis, hepatocellular carcinoma and liver-related mortality among HBV-infected individuals [1, 11, 12]. There is conflicting evidence on whether HBV increases the rate of HIV progression [10, 13].

Over 75,000 Canadians live with HIV/AIDS and 6–8% are HBV co-infected [14]. Data specific to HIV-HBV co-infection in Canada is sparse. Pittman et al. (2015) demonstrated a 5.5% HBV prevalence in Northern Albertan HIV-positive patients [1]. Co-infected males tended to be White, and females were primarily Black and from HIV-endemic countries or Indigenous. A 2013 Ontario-based HIV cohort study suggested that HIV-HBV co-infected patients were more likely to be male and MSM [4]. While these studies provide important regional information on HIV-HBV co-infection, they cannot be generalized across Canada.

There is currently no Canadian-wide demographic or clinical data focusing on this population. To address this knowledge gap, we utilized a national cohort (Canadian Observational Cohort; CANOC) [15] of HIV-infected patients to explore the prevalence, demographic characteristics, risk factors for exposure, and clinical characteristics of HIV-HBV co-infection in Canada. We specifically evaluated predictors of liver fibrosis before and after initiating ARV therapy as well as mortality.

## Methods

CANOC is an interprovincial collaboration of eight cohorts from British Columbia (BC), Quebec, and Ontario. Each site conducts data collection with local research ethics approval. Our participation in this study was submitted to and approved by the Ottawa Health Science Network Research Ethics Board (2010673-01H). Eligible participants were previously ARV treatment naïve HIV-infected patients who initiated combination ARV between January 1, 2000 and December 14, 2014 [15].

For this analysis we included only those participants from the CANOC cohort with complete HBV infection status and complete liver enzyme data, required for AST to Platelet Ratio Index (APRI) calculation. Baseline was defined as any time within 1 year prior to initiating first ARV therapy. Baseline values for CD4 T cell count and HIV viral load was obtained within 6 months prior to first ARV dosing date. End of follow-up period was defined as any time within 1 year before the patient’s last follow date or December 31, 2014.

Participants were classified as ‘HBV co-infected’ if HBV surface antigen positive, HBV DNA positive, and/or by physician report. The primary outcome, liver fibrosis stage, was calculated by the APRI, a validated non-invasive marker of fibrosis [16–18]. APRI was calculated using [AST/ (upper limit of normal AST)/platelets] × 100] [19]. The AST upper limit of normal was set at 40 IU/L. APRI ratios ≤0.50, 0.51–1.49, and ≥ 1.50 were used to define minimal, intermediate, and clinically relevant fibrosis, respectively [17].

Demographic variables of interest included age, sex, ethnicity, province of residence, alive/deceased, as well as MSM and PWID status. HCV infection status was ascertained by serology, PCR, and/or physician report. AIDS-defining illnesses (ADI) were based on established criteria [20]. HIV viral suppression was defined as two consecutive viral load < 50 copies/ml measured at least 30 days apart since the first naïve ARV date. HIV viral rebound included those who achieved viral suppression but went on to have 2 consecutive VL > 200 copies/ml measured at least 30 days apart [1, 10–12, 21].

Demographic and clinical characteristics were compared between HIV-HBV co-infected and HIV-infected groups using chi-square or Fisher exact tests for categorical variables and Wilcoxon’s Rank Sum test for continuous variables. Logistic regression models were developed to assess the association between HBV infection status and hepatic fibrosis, and to determine predictors of hepatic fibrosis at baseline and at end of follow-up. Variables that were statistically significant (*p* < 0.05) were then adjusted for in multivariable models.

## Results

The final analysis set consisted of 2419 participants of which 8% (*n* = 199) were HIV-HBV co-infected. Initially, a total of 10,477 HIV positive participants were identified in the CANOC cohort. Participants from one cohort site (*n* = 1188) were excluded from analysis because of possible HBV misclassification concerns. Additionally, 2476 were excluded due to unknown HBV status and 4394 did not have AST data at both baseline and end of study. A comparison of the baseline characteristics study between those included in our study cohort and these excluded participants did not reveal any clinically relevant differences that we believe would impact our findings (see Additional file 1: Table S1).

Age, sex, ethnicity, MSM, and province of residence were similar between HIV-infected and HIV-HBV co-infected groups (Table 1). HIV-HBV co-infected participants were more likely to acquire HIV by injection drug use (28% vs. 21%, *p* = 0.03) and to be HCV co-infected (31% vs 23%; *p* = 0.02).

**Table 1:** Demographic characteristics participants by HBV infection status (*N* = 2419)

Demographic Characteristics	HIV *N* = 2220	HIV-HBV Co-Infection *N* = 199	p value^a^
Median Age	40 (32.0–46.0)	39 (33.0–45.0)	0.69
Male Sex	1806 (81)	164 (82)	0.71
Deceased	150 (7)	22 (11)	0.02
Ethnicity			
White	967 (44)	78 (39)	0.11
Black	408 (18)	50 (25)	
Indigenous	138 (6)	10 (5)	
Asian	147 (7)	20 (10)	
Hispanic	116 (5)	9 (5)	
Other	82 (4)	5 (3)	
Unknown	362 (16)	27 (14)	
Province			
BC	946 (43)	88 (44)	0.49
ON	870 (39)	70 (35)	
QC	404 (18)	41 (21)	
Risk Factors			
MSM	998 (45)	93 (47)	0.63
PWID	467 (21)	55 (28)	0.03

*BC* British Colombia, *ON* Ontario, *QC* Quebec, *MSM* Men who have sex with men, *PWID* people who inject drugs, *ADI* AIDS defining illness, *ARV* anti-retroviral therapy

^a^Data shown are frequencies and proportions for categorical variables and median and interquartile ranges for continuous variables. *p* values for categorical variables were calculated using chi-square or Fisher exact tests and for continuous variables were calculated using Wilcoxon’s Rank Sum tests

^b^‘No ADI ever’ refers to no recorded ADI’s during study period. ‘None before/or first ARV date’ refers to no recoded ADI prior to study enrollment

The median baseline CD4 T cell count was 188 cells/ml in HIV-HBV co-infected participants (IQR: 120–360) and 235 cells/ml in HBV-negative participants (IQR: 85–294) (*p* = 0.0002) (Table 2). The proportion with an AIDS-defining illness (ADI) on or before the first ARV treatment date was also higher in HIV-HBV co-infected patients (28% vs. 20% *p* = 0.01). The median duration of ARV therapy exposure was longer in the HIV-HBV co-infected group (5.97 years; IQR: 3.11–9.94 years) compared to HIV-infected individuals (5.01 years; IQR: 2.50–8.75 years) (*p* = 0.003). The baseline and end-of-follow up HIV viral load levels were similar between groups.

**Table 2:** HIV-related characteristics of HIV-infected participants by HBV infection status (*N* = 2419)

Clinical Characteristics	HIV N = 2220	HIV-HBV Co-Infected N = 199	p value^a^
Baseline HIV viral load (Log10 copies/mL)			
< 4	332 (15)	30 (15)	0.87
4–5	1011 (46)	94 (47)	
> 5	877 (40)	75 (38)	
End of follow-up HIV viral load (Log10 copies/mL)			
< 4	2104 (95)	193 (97)	0.38
4–5	69 (3)	4 (2)	
> 5	47 (2)	2 (1)	
Baseline CD4 T cell count (cells/mm^3^)	235 (120–360)	188 (85–294)	0.0002
Baseline CD4 T cell count category (cells/mm^3^)			
≤ 199	897 (40)	105 (53)	0.002
200–349	720 (32)	60 (30)	
350–499	367 (17)	24 (12)	
> 500	236 (11)	10 (5)	
Baseline AIDS-Defining Illness^b^			
≥ 1	446 (20)	55 (28)	0.01
None	1774 (80)	144 (72)	
Years on ARV therapy	5.01 (2.50–8.75)	5.97 (3.11–9.94)	0.003
HIV Suppressed since FARVDT	2040 (92)	190 (96)	0.07
HIV Rebound (since first VS)	481 (24)	56 (29)	0.07

*VS* virological suppression, *FARVDT* first naïve ARV date

^a^Data shown are frequencies and proportions for categorical variables and median and interquartile ranges for continuous variables. p values for continuous variables were calculated using chi-square or Fisher exact tests and for continuous variables were calculated using Wilcoxon’s Rank Sum tests

^b^AIDS-defining illness present prior to or before first naïve ARV date

HIV-HBV co-infected patients had higher median APRI scores at baseline (0.50 vs. 0.37, *p* < 0.0001) (Table 3). Overall, 6.5% (*n* = 159) participants had clinically relevant fibrosis (APRI≥1.5) at baseline. This proportion was higher (20%) in those with HIV-HBV co-infection. While the rate of HIV RNA viral load suppression did not differ by APRI score, those with clinically relevant fibrosis were less likely to rebound following viral suppression (69% vs. 79%, *p* = 0.05).

**Table 3:** Liver-Specific characteristics of HIV-infected participants by HBV infection status (*N* = 2419)

Clinical Characteristics	HIV N = 2220	HIV-HBV Co-Infected N = 199	p value^a^
Hepatitis C co-infection	520 (23)	61 (31)	0.02
Baseline AST (IU/L)	29 (23–41)	36 (26–62)	< 0.0001
Baseline AST category			
Normal (10–40 IU/L)	1649 (74)	120 (60)	< 0.0001
Elevated (> 40 IU/L)	571 (26)	79 (40)	
End of follow-up AST	25 (20–33)	26 (20–38)	0.14
End of follow-up AST category			
Normal (10–40 IU/L)	1855 (84)	152 (76)	0.01
Elevated (> 40 IU/L)	365 (17)	47 (24)	
Baseline APRI	0.37 (0.27–0.59)	0.50 (0.35–1.04)	< 0.0001
Baseline APRI Fibrosis category			
≤ 0.5 (minimal)	1501 (68)	99 (50)	< 0.0001^b^
0.51–1.49 (moderate)	593 (27)	68 (34)	
1.50–1.99 (significant)	34 (2)	8 (4)	
≥ 2.0 (advanced/cirrhosis)	92 (4)	24 (12)	
End of follow-up APRI	0.30 (0.22–0.43)	0.32 (0.23–0.56)	0.03
End of follow-up APRI Fibrosis category			
≤ 0.5 (minimal)	1831 (82)	145 (73)	0.005^b^
0.51–1.49 (moderate)	302 (14)	39 (20)	
1.50–1.99 (significant)	24 (1)	3 (2)	
≥ 2.0 (advanced/cirrhosis)	63 (3)	12 (6)	

*AST* aspartate aminotransferase, *APRI* aspartate aminotransferase to platelet ratio index, *VS* virological suppression, *FARVDT* first naïve ARV date

^a^Data shown are frequencies and proportions for categorical variables and median and interquartile ranges for continuous variables. p values for continuous variables were calculated using chi-square or Fisher exact tests and for continuous variables were calculated using Wilcoxon’s Rank Sum tests

^b^*p* values were calculated using Kruskal-Wallis rank tests

Individuals were more likely to have clinically relevant fibrosis at baseline if they were HBV (OR 3.08, 95% CI: 1.99–4.78) or HCV co-infected (OR 4.55, 95% CI: 3.26–6.35) after adjusting for demographic characteristics (Table 4). Clinically relevant fibrosis was also associated with older age at first ARV date (OR 1.02, 95% CI: 1.00–1.04) and baseline HIV RNA level > 5 log10 copies (OR 2.15, CI: 1.16–3.97).

**Table 4:** Logistic regression model showing factors associated with clinically relevant fibrosis at baseline (N = 2419)

	APRI> 1.5 (clinically relevant fibrosis) at baseline					
	Univariate Analysis			Multivariable Analysis		
Variable	OR	95% CI	Wald p	OR	95% CI	Wald p
Hepatitis B						
HBV negative		Reference			Reference	
HBV positive	3.18	2.10–4.84	< 0.0001	3.08	1.99–4.78	< 0.001
Hepatitis C						
HCV negative		Reference			Reference	
HCV positive	4.77	3.43–6.63	< 0.0001	4.55	3.26–6.35	< 0.001
Baseline AIDS						
No ADI ever		Reference				
None before/at FARVDT	1.49	0.74–2.98	0.44		–	
≥ 1 before/at FARVDT	1.63	0.77–3.46				
Race						
White		Reference				
Black	0.40	0.23–0.70				
Indigenous	1.13	0.61–2.09				
Asian	0.67	0.33–1.36	0.01		–	
Hispanic	0.29	0.09–0.93				
Other	1.35	0.66–2.80				
Unknown	0.88	0.56–1.38				
Birth sex						
Female		Reference				
Male	1.06	0.70–1.62	0.78		–	
Province						
BC		Reference				
ON	0.47	0.32–0.68	< 0.0001		–	
QC	0.44	0.26–0.72				
Years on ARV	1.02	0.98–1.06	0.37			
Age at first ARV	1.02	1.01–1.04	0.003	1.02	1.00–1.04	0.01
MSM	0.63	0.46–0.88	0.006		–	
PWID	3.47	2.49–4.82	< 0.0001		–	
Baseline HIV viral load (Log10 copies/mL)						
< 4		Reference			Reference	
4–5	1.68	0.91–3.08	0.004	1.49	0.80–2.77	0.21
> 5	2.46	1.35–4.48		2.15	1.16–3.97	0.02
Baseline CD4 count (cells/mm^3^)						
> 500		Reference				
350–499	1.60	0.70–3.70				
200–349	1.82	0.85–3.92	0.0007			
≤ 100	2.76	1.32–5.77				

*FARVDT* first naïve ARV date, *MSM* Men who have Sex with Men, *PWID* People Who Infect Drugs, *BC* British Colombia, *ON* Ontario, *QC* Quebec

Variables considered in the multivariate model included: hepatitis B, hepatitis C, race, province, age at first ARV treatment, MSM, baseline HIV viral load, baseline CD4 count. Due to co-linearity with hepatitis C, PWID was not included in the final model

APRI scores declined once on ARV therapy, especially among HIV-HBV co-infected patients. Median APRI scores were only slightly greater among co-infected patients at end of follow-up (0.32 vs. 0.30, *p* = 0.03) (Table 5). By multivariable analysis, HIV-HBV co-infection did not predict clinically relevant fibrosis at the end of follow-up. Clinically relevant fibrosis at end of follow-up was predicted by HCV co-infection, lower baseline CD4 T cell counts and increased HIV RNA level at the end of study. We tested for HBV effect modification by including interaction terms in logistic regression models, and none were identified (data not shown).

**Table 5:** Logistic regression model showing factors associated with clinically relevant fibrosis at end of study follow-up (N = 2419)

	APRI> 1.5 (clinically relevant fibrosis) at end of follow-up					
	Univariate Analysis			Multivariable Analysis		
Variable	OR	95% CI	Wald p	OR	95% CI	Wald p
Hepatitis B						
Never co-infected		Reference			–	
Ever co-infected	2.00	1.13–3.53	0.02			
Hepatitis C						
Never co-infected		Reference			Reference	
Ever co-infected	7.03	4.61–10.74	< 0.0001	6.35	4.12–9.79	< 0.0001
Baseline AIDS						
No ADI ever		Reference				
None before/at FARVDT	1.72	0.69–4.31	0.41		–	
≥ 1 before/at FARVDT	1.95	0.73–5.19				
Race						
White		Reference				
Black	0.28	0.12–0.65				
Indigenous	1.83	0.95–3.54				
Asian	0.77	0.33–1.84	0.009		–	
Hispanic	0.51	0.16–1.67				
Other	1.00	0.35–2.84				
Unknown	1.31	0.78–2.18				
Birth sex						
Female		Reference			–	
Male	0.69	0.43–1.10	0.12			
Province						
BC		Reference				
ON	0.44	0.28–0.70	0.0001		–	
QC	0.34	0.17–0.67				
Years on ARV	0.97	0.92–1.02	0.26		–	
Age at first ARV	1.01	0.99–1.02	0.21		–	
MSM	0.34	0.26–0.60	< 0.0001		–	
PWID	5.05	3.37–7.56	< 0.0001		–	
Baseline HIV viral load (Log10 copies/mL)			0.17			
< 4		Reference			–	
4–5	1.46	0.72–2.93				
> 5	1.87	0.94–3.74				
Follow-up HIV viral load (Log10 copies/mL)						
< 4		Reference			Reference	
4–5	3.33	1.54–7.16	< 0.0001	2.27	1.01–5.10	< 0.0001
> 5	8.80	4.41–17.43		7.33	3.46–15.51	
Baseline CD4 count (cells/mm^3^)						
≤ 100		Reference			Reference	
200–349	0.55	0.35–0.86		0.62	0.38–0.99	
350–499	0.15	0.06–0.42	0.0001	0.18	0.07–0.51	0.004
> 500	0.37	0.16–0.86		0.55	0.23–1.32	

*FARVDT* first naïve ARV date, *MSM* Men who have Sex with Men, *PWID* People Who Infect Drugs

*BC* British Colombia, *ON* Ontario, *QC* Quebec

Variables considered in the multivariate model included: hepatitis B, hepatitis C, race, province, age at first ARV treatment, MSM, follow-up HIV viral load, baseline CD4 count. Due to co-linearity with hepatitis C, PWID was not included in the final model

The overall mortality rate by end of follow-up was 7.1%. HIV-HBV co-infected participants had a higher mortality rate (11% vs. 7%, *p* = 0.02). The mortality rate among participants with clinically relevant fibrosis at baseline was higher (23% vs 6%, *p* < 0.0001).

## Discussion

Our study is among the first to describe the epidemiology of HIV-HBV co-infection in Canada at the time of ARV initiation. Our cohort included approximately 20% of all HIV-infected individuals in Canada and is largely representative of those initiating ARV since 2000 [15]. The prevalence of HIV-HBV co-infection in our analysis was 8%, which is consistent with previously established co-infection rates in the general Canadian population and reported by other North American HIV cohorts [1, 10–12, 21–23]. This is slightly greater than the 5.5% rate determined by the Northern Alberta cohort, which likely represents inter-regional differences in co-infection prevalence [1].

Despite the existence of universal HBV vaccination programs in Canada, the number of reported cases of HBV infection continues to increase, driven primarily by non-Canadian born immigrants [1, 14]. Many of the Canadian-born patients in our cohort were likely too old to have received HBV immunization in their youth as this practice in Canada did not begin until the 1990s [24–26]. There were no statistically significant differences in the racial distribution of the HIV-HBV co-infected group compared to HIV mono-infected cohort participants. We acknowledge that the large proportion of missing data on race may have affected these results. However, our findings are in keeping with the Northern Alberta cohort, in which the majority of HIV-HBV co-infected males were White, and females were mainly Black or Indigenous [1]. The rate of acute HBV in Indigenous persons in Canada is approximately three times that of non-Indigenous persons [14, 27]. However, in our cohort of HIV-infected participants, the rate of HBV co-infection among Indigenous people was comparable to the general population.

The high prevalence of MSM and PWID in our cohort is in keeping with other studies in areas with low HBV endemicity [10, 11, 28]. The prevalence of HCV infection and injection drug use in our cohort was higher among participants with HIV-HBV co-infection compared to HIV infected patients, which is consistent with other viral hepatitis-HIV co-infection studies [11, 29, 30]. The proportion of MSM did not differ between HIV-infected and HIV-HBV co-infected groups. While this is in alignment with Pittman et al, who found no independent association between MSM and HBV co-infection, [1] other HIV cohorts have reported a higher proportion of MSM among patients who are HIV-HBV co-infected [12, 28]. We speculate our data may represent a shift in epidemiology in which HBV infection is more related to injection drug use rather than sexual transmission among the HIV-infected population in Canada.

HBV infection is often asymptomatic and as many as 30% of patients have no identified risk factors for exposure, thereby making screening necessary to identify HBV infection [14, 31, 32]. As 10% of patients in CANOC had unknown HBV status, there is a continuing need to bolster screening efforts in this population [33]. Knowledge of HBV co-infection is also highly relevant to decisions related to ARV selection.

We demonstrated lower baseline CD4 T cell counts and an increased rate of ADI among HIV-HBV co-infected patients compared to HIV-infected patients. Our observation of lower baseline CD4 T cell counts in HIV-HBV co-infected patients has been replicated in some, [34–36] but not all, analyses [13, 35, 37, 38]. Lower CD4 counts in HIV-HBV co-infected individuals have been associated with higher levels of HBV viral replication (HBV DNA > 200,000 IU/mL) and may increase the risk of developing hepatocellular carcinoma [12, 39]. HIV-HBV co-infected individuals had a higher prevalence of HCV co-infection. HCV co-infection may negatively influence CD4 T cell counts in HIV-infected patients [22, 40, 41]. This may have contributed to a lower baseline CD4 T cell count in the HBV-HIV co-infected group. However, several studies that controlled for ARV therapy, baseline clinical characteristics, and other potential confounders did not demonstrate any differences in ADI or CD4 T cell recovery [42–45]. It is also plausible that CD4 T cell counts may have been reduced by splenic sequestration [46–48].

Clinically relevant fibrosis at baseline was prevalent in 20% of HIV-HBV co-infected participants. We demonstrated that HBV co-infection and older age were associated with baseline clinically relevant fibrosis. This is in keeping with other studies that have used non-invasive measures to determine the extent of fibrosis in HIV-HBV co-infected patients [36, 49, 50]. While fibrosis scores improved in all participants during follow-up, there was a greater regression among HIV-HBV co-infected patients. At the end of follow-up (median 5.97 years among HIV-HBV co-infected participants) HBV co-infection was no longer independently associated with fibrosis. This is consistent with previous studies of 1–3 years involving HBV-HIV co-infected patients demonstrating that HBV-active antiretroviral therapy reduces inflammation and fibrosis by suppressing HBV infection [51–55]. Our results suggest that fibrosis in HBV-HIV co-infected patients may continue to normalize years after initiating ARV therapy.

Patients who had high baseline HIV RNA levels were more likely to have clinically relevant fibrosis, both at baseline and at end of follow-up. Baseline CD4 T cell count was not a predictor of clinically relevant fibrosis by multivariable analysis. The influence of HIV on progression of HBV-related liver disease has been attributed to a number of mechanisms including direct infection of hepatic cells, [56] indirectly through ARV hepatotoxicity [57, 58], CD4 T cell depletion causing cytokine deregulation and inflammation [59] and/or impaired T cell response [60]. The demonstrated association between fibrosis and HIV RNA level prior to ARV therapy, but not CD4 T cell count, lends support to a direct effect of HIV infection on fibrosis that is distinct from an effect on T-cell response or drug toxicity.

Several studies have suggested an inverse correlation between CD4 T cell count and fibrosis in HBV-HIV co-infection, [46–48] as well as an association between low baseline CD4 T cell counts and increased liver-related mortality [7, 12]. Our analysis did not identify an association between low CD4 T cell counts and clinically relevant fibrosis at baseline. This may suggest that the decreased CD4 T cell counts were not a cause of fibrosis but rather a result of splenic sequestration of leukocytes due to portal hypertension [46–48]. We did demonstrate that low baseline CD4 T cell counts were associated with fibrosis at end of follow-up, which is consistent with findings of a previous prospective study of HIV-HBV co-infected patients followed for a median of five years [61]. While it would be of interest to compare fibrosis with CD4 T cell counts at end of follow-up, CD4 T cells counts were only recorded at baseline.

We observed higher mortality among HIV-HBV co-infected participants compared to HIV-infected individuals. We suspect that this is due in part to a consequence of excess liver-disease related mortality [7–10, 62]. While we could not confirm this as liver-specific mortality is not captured in CANOC, we did demonstrate increased fibrosis among HIV-HBV co-infected participants, and that participants with APRI> 1.5 were more likely to be deceased at follow-up. This is consistent with previous work [63].

Certain limitations are acknowledged. There was a large proportion of missing data due to incomplete or unknown HBV infection testing and lack of baseline APRI measurements. Although we did not identify any large differences between our analysis set and excluded patients it is possible that unrecognized biases may have influenced our findings. The lower proportion of PWID in our analysis sample may have biased the proportion with HBV co-infection as injection drug use is a known risk factor for HBV exposure. As APRI scores were our primary study outcome, we opted for complete case analysis to address missing data concerns. We did not have access to data including HBV infection date, repeated HBV infection tests, and HBV treatment. Alcohol consumption information, a potentially important confounder for fibrosis evaluation, was not available. We cannot necessarily generalize our findings to patients starting ARV prior to January 2000. Our study includes data from three provinces. Efforts to include more Canadian regions have been made and the next CANOC dataset will include greater regional representation, which will increase the generalizability of this cohort.

## Conclusion

Our evaluation demonstrates a HBV co-infection prevalence of 8% in HIV-infected patients in BC, Ontario, and Quebec. HIV-HBV co-infected participants were more likely to have a history of injection drug use and to be HCV co-infected. HIV-HBV co-infected participants had lower CD4 T cell counts at baseline and higher levels of hepatic fibrosis scores compared to HIV-infected patients. However, among patients receiving ARV therapy, there was no clinically significant difference in fibrosis at the end of follow up between HIV-HBV co-infected and HIV-infected individuals. Our results help illuminate the clinical and demographic characteristics of this under-studied population. The prevalence and clinical correlates of HIV-HBV co-infection, namely reduced CD4 T cell count and fibrosis, reinforce the need for routine HBV screening among HIV-infected patients.

## Supplementary information

**Additional file 1: Table S1.** Demographic characteristics participants by inclusion status (*N* = 10,447).

## Data Availability

The datasets generated and/or analyzed during the current study are not publicly available due privacy policies but are available from the corresponding author on reasonable request.

## Abbreviations

ADI: AIDS defining illness
APRI: AST to Platelet Ratio Index
ARV: antiretroviral therapy
AST: aspartate aminotransferase
BC: British Columbia
CANOC: Canadian Observational Cohort Collaboration
CD4: cluster of differentiation 4
CI: confidence interval
DNA: deoxyribonucleic acid
HBV: Hepatitis B virus
HCV: Hepatitis C virus
HIV: Human Immunodeficiency Virus
IQR: interquartile range
MSM: men who have sex with men
OR: odds ratio
PWID: people who inject drugs
RNA: ribonucleic acid
VL: viral load
