# Supplementary material for: Characteristics and outcomes of antiretroviral-treated HIV-HBV co-infected patients in Canada?
Source: BMC Infect Dis. 2019 Nov 21;19:982. doi: 10.1186/s12879-019-4617-8 (PMC6873547; doi:10.1186/s12879-019-4617-8)
Supplement: Supplementary file 1 — Additional file 1: Table S1. Demographic characteristics participants by inclusion status (N = 10,447). [file 12879_2019_4617_MOESM1_ESM.docx]

| **Demographic** | **Included in analyses** | **Not Included in analyses** | **p value^a^** |
| --- | --- | --- | --- |
| **Characteristics** |  |  |  |
|  | **N=2,419** | **N=8,028** |  |
| **Median Age** | 39 (32-46) | 40 (33-47) | 0.26 |
| **Male Sex** | 1,957 (81) | 6581 (82) | 0.28 |
| **Deceased** | 172 (7) | 789 (10) | <0.001 |
|  |  |  |  |
| **Ethnicity** |  |  |  |
| White | 1045 (43) | 2659 (33) | <0.001 |
| Black | 458 (19) | 633 (8) |  |
| Indigenous | 148 (6) | 555 (7) |  |
| Asian | 167 (7) | 251 (3) |  |
| Hispanic | 125 (5) | 246 (3) |  |
| Other | 87 (4) | 244 (3) |  |
| Unknown | 389 (16) | 3440 (43) |  |
|  |  |  |  |
| **Risk Factors** |  |  |  |
| MSM | 1328 (55) | 3370 (53) | 0.21 |
| PWID | 522 (22) | 1725 (26) | <0.001 |
|  |  |  |  |

**Supplementary Table 1. Demographic characteristics participants by inclusion status (N=10,447)**

MSM: Men who have sex with men; PWID: people who inject drugs

^a^ Data shown are frequencies and proportions for categorical variables and median and interquartile ranges for continuous variables. p values for categorical variables were calculated using chi-square or Fisher exact tests and for continuous variables were calculated using Wilcoxon’s Rank Sum tests.
